# Chromatin Changes in Phytochrome Interacting Factor-Regulated Genes Parallel Their Rapid Transcriptional Response to Light

**DOI:** 10.3389/fpls.2022.803441

**Published:** 2022-02-17

**Authors:** Eduardo González-Grandío, Simón Álamos, Yu Zhang, Jutta Dalton-Roesler, Krishna K. Niyogi, Hernán G. García, Peter H. Quail

**Affiliations:** ^1^Department of Plant and Microbial Biology, University of California, Berkeley, Berkeley, CA, United States; ^2^Plant Gene Expression Center, Agricultural Research Service, US Department of Agriculture, Albany, CA, United States; ^3^Feedstocks Division, Joint BioEnergy Institute, Emeryville, CA, United States; ^4^Howard Hughes Medical Institute, University of California, Berkeley, Berkeley, CA, United States; ^5^Molecular Biophysics and Integrated Bioimaging Division, Lawrence Berkeley National Laboratory, Berkeley, CA, United States; ^6^Department of Molecular and Cell Biology, University of California, Berkeley, Berkeley, CA, United States; ^7^Department of Physics, University of California, Berkeley, Berkeley, CA, United States; ^8^California Institute for Quantitative Biosciences (QB3), University of California, Berkeley, Berkeley, CA, United States; ^9^Biophysics Graduate Group, University of California, Berkeley, Berkeley, CA, United States

**Keywords:** photomorphogenesis, histone acetylation, transcriptional regulation, phytochrome interacting factor (PIF), chromatin modification and gene reprogramming

## Abstract

As sessile organisms, plants must adapt to a changing environment, sensing variations in resource availability and modifying their development in response. Light is one of the most important resources for plants, and its perception by sensory photoreceptors (e.g., phytochromes) and subsequent transduction into long-term transcriptional reprogramming have been well characterized. Chromatin changes have been shown to be involved in photomorphogenesis. However, the initial short-term transcriptional changes produced by light and what factors enable these rapid changes are not well studied. Here, we define rapidly light-responsive, Phytochrome Interacting Factor (PIF) direct-target genes (LRP-DTGs). We found that a majority of these genes also show rapid changes in Histone 3 Lysine-9 acetylation (H3K9ac) in response to the light signal. Detailed time-course analysis of transcript and chromatin changes showed that, for light-repressed genes, H3K9 deacetylation parallels light-triggered transcriptional repression, while for light-induced genes, H3K9 acetylation appeared to somewhat precede light-activated transcript accumulation. However, direct, real-time imaging of transcript elongation in the nucleus revealed that, in fact, transcriptional induction actually parallels H3K9 acetylation. Collectively, the data raise the possibility that light-induced transcriptional and chromatin-remodeling processes are mechanistically intertwined. Histone modifying proteins involved in long term light responses do not seem to have a role in this fast response, indicating that different factors might act at different stages of the light response. This work not only advances our understanding of plant responses to light, but also unveils a system in which rapid chromatin changes in reaction to an external signal can be studied under natural conditions.

## Introduction

One of the most drastic changes during plant development is deetiolation, the switch from skotomorphogenesis (development in the dark) into photomorphogenesis (development in the light). This change not only implies switching from heterotrophy to autotrophy, but also includes several developmental changes such as reduced hypocotyl elongation, opening of the apical hook and greening of cotyledons (Schafer and Nagy, 2006; Franklin and Quail, 2010). Light signals that trigger deetiolation are perceived by photoreceptors. In Arabidopsis, phytochrome A (phyA) and phyB are the main sensors that regulate early photomorphogenesis (Franklin and Quail, 2010). Upon photoactivation, the active phy is translocated from the cytoplasm into the nucleus where it physically interacts with Phytochrome Interacting Factors (PIFs), inducing their rapid transphosphorylation, ubiquitination and proteasome-mediated degradation of the phy-PIF complex (Bauer et al., 2004; Nagatani, 2004; Al-Sady et al., 2006; Jiao et al., 2007; Bae and Choi, 2008; Pfeiffer et al., 2012; Ni et al., 2013, 2014, 2017). PIFs are a subfamily of bHLH transcription factors, comprising eight members in *Arabidopsis thaliana*. PIF1, PIF3, PIF4, and PIF5 (known as the “PIF quartet”) have a central role in maintaining the transcriptional program that underlies skotomorphogenic development (Leivar et al., 2008; Shin et al., 2009). The quadruple mutant for these four PIFs (*pifq*) displays a phenotype in total darkness that strongly resembles that of normal light-grown wild-type seedlings (Leivar et al., 2008; Shin et al., 2009).

To identify PIF-direct target genes (PIF-DTGs), our group analyzed the genome-wide binding profile of each of the PIF quartet members by Chromatin Immunoprecipitation-sequencing (ChIP-seq), and the corresponding transcriptomic profile of dark-grown wild type, *pifq*, single *pif* and triple *pif* mutants, by RNA sequencing (RNA-seq; Hornitschek et al., 2012; Zhang et al., 2013; Pfeiffer et al., 2014). Integration of both datasets allowed the identification of 338 PIF-DTGs, genes whose promoter region is bound by one or more PIF quartet members at a G-box or PIF Binding Element, and whose transcript levels are misregulated in *pifq* mutant plants grown in the dark (Pfeiffer et al., 2014). General transcriptional reprogramming that results in photomorphogenesis, occurs upon light exposure of dark-grown plants, as a consequence of PIF degradation (Leivar et al., 2009). There is evidence for antagonistic competition between the PIFs and HY5 for G-box binding in the promoter of the *PSY* gene during early light-induced expression (Toledo-Ortiz et al., 2014). However, the initial dynamics of light-induced transcriptional changes of PIF-DTGs as a whole has not been studied.

In eukaryotes, chromatin structure modification is a key factor of transcription regulation (Venkatesh and Workman, 2015). Among the many histone post-translational modifications, acetylation seems to play a major role in this process (Jiang et al., 2020). Histone acetylation, catalyzed by histone acetyltransferases (HATs), has been associated with transcriptional activation, while histone deacetylation by histone deacetylases (HDACs) is linked to transcriptional repression (Pandey et al., 2002). Whether the role of histone modifications in transcriptional regulation is causal or consequential is not well understood (Henikoff and Shilatifard, 2011; Morgan and Shilatifard, 2020). Previous research has established that histone acetylation plays an essential role during photomorphogenesis (Barneche et al., 2014). Transcriptional regulation and development of light-grown plants is greatly altered in Histone 3 Lysine 9 acetylation (H3K9ac) deposition (HAG1/GCN5 and HAF2/TAF1) and removal mutants (HDA19/HD1) (Bertrand et al., 2005; Benhamed et al., 2006; Guo et al., 2008). Recent evidence suggests that PIF-exerted transcriptional regulation might be executed by altering the histone modification landscape. It has been described that PIF1 and PIF3 directly interact with HDA15 in the dark to repress the expression of germination and chlorophyll biosynthesis genes, respectively (Liu et al., 2013; Gu et al., 2017). PIF7 has also been shown to induce H3K9ac deposition on its downstream genes in response to changes in light quality (Peng et al., 2018; Martínez-García and Moreno-Romero, 2020; Willige et al., 2021). HY5, a key photomorphogenesis transcription factor also interacts with HDA15 and decreases histone acetylation of genes involved in hypocotyl elongation during photomorphogenesis to repress their expression (Zhao et al., 2019). Additionally, phyB has been shown to redundantly control chromatin remodeling to inhibit the transcriptional activation of growth-promoting genes by PIFs (Kim et al., 2021). It is still unclear if these factors that control histone acetylation in dark or light-grown plants are also involved in the very initial steps after light exposure that will trigger the photomorphogenesis transcriptional program.

Here, to explore the potential role of chromatin remodeling as an intermediary in light-triggered regulation of PIF-DTGs, we focused on H3K9 acetylation as a mark of transcriptionally active genes. We first identified rapid light-responding PIF-DTGs by comparing the transcriptomic profile of dark-grown seedlings with those of dark-grown *pifq* mutants and dark-grown seedlings after a short red-light treatment. We also profiled genome-wide H3K9ac localization in these plants. We found that the majority of light-responsive PIF-DTGs also showed changes in H3K9ac. We then conducted a detailed time-course analysis of mRNA and H3K9ac levels on selected light-responsive PIF-DTGs. This analysis initially suggested that the relationship between H3K9ac and transcription differs in light-repressed and light-induced PIF-DTGs. Real-time transcription initiation imaging, however, suggests instead that H3K9ac also parallels transcriptional induction in response to light.

## Results and Discussion

### Identification of Rapidly Responding, Light Regulated Phytochrome Interacting Factor-Direct Target Genes

**FIGURE 1:**
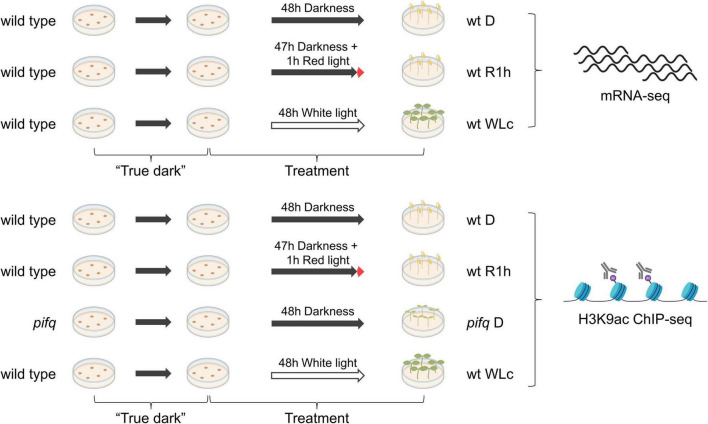
Experimental setup. Wild type or *pifq* seedlings were grown in “true dark” conditions and treated for 2 days before sample collection and processing for mRNA- or H3K9ac ChIP-sequencing (see section “Materials and Methods” for detailed experimental procedures).

In our previous work, we defined PIF-DTGs as genes that are misregulated in *pifq* mutant plants grown in the dark and whose promoter is bound by any one or more of the PIF quartet proteins [209 PIF-induced and 129 PIF-repressed-DTGs (Pfeiffer et al., 2014)]. In order to identify genes that respond most directly to changes in PIF abundance, we focused on genes whose transcript levels change rapidly in wild-type seedlings after a short exposure to red light, when the PIFs have been almost completely degraded (Bauer et al., 2004; Shen et al., 2005, 2007; Lorrain et al., 2007). For this purpose, we measured genome-wide steady state mRNA levels by RNA-sequencing in “true dark”-grown, wild-type seedlings (D) and in “true dark”-grown seedlings treated for 1 h with red light (R1h). We also measured mRNA levels in seedlings grown in continuous white light (WLc) as a reference expression profile of plants grown during an extended light regimen (Figure 1).

**FIGURE 2:**
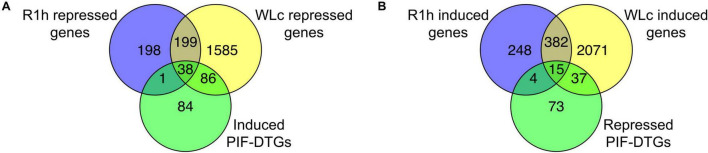
A subset of PIF-DTGs are induced or repressed after a short exposure to red light, and a larger set of genes change expression under continuous white light conditions. **(A)** 39 PIF-induced DTGs are repressed after 1 h of red light, and 86 additional genes are repressed in continuous white light, totaling 125 out of 209 PIF-induced DTGs (60%). **(B)** 19 PIF-repressed DTGs are induced after 1 h of red light, and 37 additional genes are induced in continuous white light, totaling 56 out of 129 PIF-repressed DTGs (43%). Only genes with a statistically significant (FDR < 0.05) two-fold change in expression under different treatments are compared. PIF-DTGs are defined in Pfeiffer et al. (2014) as genes whose promoter region is bound by one or more PIF quartet members at a G-box or PIF Binding Element, and whose transcript levels are misregulated in *pifq* mutant plants grown in the dark.

An initial analysis of R1h and WLc transcriptional profiles compared to D showed that the large transcriptional reprogramming occurring in WLc (4413 genes are WLc-responsive) could be triggered by a small number of genes that change rapidly in response to the first exposure to light (1085 genes are R1h-responsive). These genes do not necessarily need to be continuously activated or repressed once the transcriptional reprogramming has been initiated, our data showed that only somewhat over half of the genes (58.4%) that change initially in response to light exposure remain in that state after an extended light regime (Figure 2 and Supplementary Datasets 1, 2).

We then analyzed the response of previously identified PIF-DTGs (Pfeiffer et al., 2014) in R1h and WLc. Surprisingly, after R1h, only 19% PIF-induced PIF-DTGs were repressed, and 15% of the PIF-repressed PIF-DTGs were induced (Figure 2, Supplementary Figure 1, and Supplementary Datasets 3, 4). Previously identified PIF-DTGs that do not change rapidly in response to light could have a slower transcriptional response to PIF degradation, be indirectly regulated by them or could have been misidentified as PIF-DTGs due to non-functional PIF binding resulting in a lack of transcriptional regulation (see below). These possibilities are non-mutually exclusive and seem to be occurring. Using the WLc data, we could identify slower-responding PIF-regulated genes: 60% of PIF-induced PIF-DTGs are repressed in R1h, WLc or in both treatments, and 43% of PIF-repressed PIF-DTGs are induced in R1h, WLc or in both treatments (Figure 2). Still, some PIF-DTGs do not show altered transcription either after R1h or WLc. Their altered transcriptional levels in *pifq* mutants grown in the dark could be caused by indirect effects (they could be downstream targets of PIF-DTGs). Additionally, these genes could represent cases where PIF-binding is non-functional and does not result in transcriptional regulation of the gene downstream of the PIF-binding site, as has been reported for other transcription factors in many ChIP-seq experiments (Biggin, 2011). These results reflect the limitation of using transcriptomic profiling of constitutive mutants in conjunction with genome wide binding assays to identify transcription factor direct target genes, leading to overestimation of the actual number of direct downstream genes. Complementing these studies with short-term response assays (in this case R1h) is essential to narrow down direct targets of transcription factors.

In summary, we have identified PIF-regulated genes whose expression changes very rapidly in response to light. These genes are directly regulated by PIFs and are likely candidates to effect the initial changes downstream of light perception that will trigger the photomorphogenesis developmental plan. For convenience, we will use the term “LRP-DTGs” (for Light Responsive PIF Direct Target Genes) from here on to refer to the newly redefined genes that are PIF-DTGs (bound by PIFs in the dark, have altered mRNA levels in the *pifq* mutants in the dark) that respond rapidly to R1h (Supplementary Dataset 5). Gene ontology enrichment analysis of light-repressed/PIF-induced LRP-DTGs revealed that they are enriched in transcription factors and in proteins known to be involved in red light, far-red light and auxin responses. These genes include *IAA19*, *IAA29*, *PIL1*, *PIL2*, and *HB2*. Light-induced/PIF-repressed LRP-DTGs were also enriched in proteins known to be involved in far-red, red and blue light responses, including genes such as *SPA1*, *RPT2*, *SIGE*, and *LHCB2.4*.

### Chromatin Changes Shortly After Red-Light Exposure

To investigate the possible involvement of rapid chromatin remodeling in the PIF-exerted regulation of LRP-DTGs, we conducted genome-wide profiling of H3K9ac by ChIP-seq in dark-grown wild type and *pifq* seedlings, and R1h-treated wild type seedlings. We also profiled H3K9ac in wild type seedlings grown in WLc for comparison as we did for transcriptome profiling (Figure 1). We chose H3K9ac because it is a histone modification that has been shown to be associated with transcriptionally active genes and also involved in long-term transcriptional regulation in continuous white light (Benhamed et al., 2006; Guo et al., 2008; Gates et al., 2017).

We identified H3K9ac peaks in all samples, and differentially quantified them comparing wt D vs. R1h, wt D vs. wt WLc, and wt D vs. *pifq* D. We found that the majority of the H3K9ac peaks mapped slightly downstream of the transcriptional start site region irrespective of light treatment, in concordance with previously published results for genome-wide H3K9ac profiles (Supplementary Figure 2; Charron et al., 2009). In dark-grown *pifq*, 7089 genes had statistically significantly higher H3K9ac levels than in wild type, while 900 genes had statistically lower H3K9ac levels (Supplementary Dataset 6). In WLc samples, 6797 genes had higher H3K9ac levels and 2505 genes had lower levels (Supplementary Dataset 7). These data are consistent with a change in the chromatin profile associated with photomorphogenic-like development in dark-grown *pifq* plants, where light-responsive genes become activated in the dark in the absence of the PIF quartet. In fact, clustering analysis revealed that the H3K9ac profile of *pifq* D is more similar to that of WLc-grown wild type than to dark-grown wild type or R1h-treated (Supplementary Figure 3).

After 1 h of red light, 995 genes had statistically significant higher H3K9ac levels than in dark-grown seedlings while 327 genes had lower H3K9ac (Supplementary Dataset 8). These results indicate that H3K9ac modification occurs only in a small fraction of light-regulated genes initially after light-exposure, as is the case for mRNA levels. H3K9ac levels change in a larger number of genes as light-exposure is sustained over longer periods, in a similar fashion to transcriptional changes. In every condition tested, H3K9ac levels generally correlated with mRNA levels (Pearson’s correlation, *r* = 0.68 for *pifq* vs. D, *r* = 0.67 for R1h vs. D and *r* = 0.8 for WLc vs. D), for genes with a statistically significant two-fold (or greater) change in mRNA and H3K9ac levels (Supplementary Figure 4).

**FIGURE 3:**
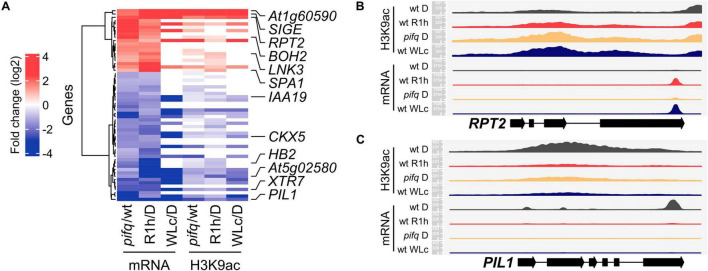
H3K9ac levels change in light-responsive PIF-DTGs (LRP-DTGs) both in *pifq* and in response to red light. **(A)** Heat-map showing the changes in mRNA and H3K9ac levels comparing dark-grown pifq versus wild type, red-light treated (R1h) versus dark-grown wild type (D), and continuous white light-grown (WLc) vs. dark grown wild-type seedlings. All the PIF-DTGs that showed statistically significant two-fold change in mRNA levels in the same direction in pifq vs. wt and R1h vs. D are shown. Six light-induced/PIF-repressed and six light-repressed/PIF-induced genes for further analyses are highlighted. **(B)** Read mapping profile of H3K9ac ChIP-seq and RNA-seq in RPT2, a light-induced LRP-DTG. **(C)** Read mapping profile of H3K9ac ChIP-seq and RNA-seq in PIL1, a light-repressed LRP-DTG. For each gene, 1000 bp upstream and 250 bp downstream of their representative transcript are shown. Read count is scaled independently for each gene, and for mRNA and H3K9ac levels. RNA-seq data from pifq seedlings and their corresponding wild-type control were obtained from Zhang et al. (2013). Note that RNA-seq was performed on 3′-end purified mRNA.

To explore whether the PIF quartet are involved in the rapid red-light-induced chromatin changes, we focused our analysis of H3K9ac changes in LRP-DTGs in dark-grown *pifq* mutants, and in R1h-exposed wild type samples. We found that a high proportion of LRP-DTGs undergo H3K9ac changes that are associated with red-light induced PIF degradation (Figure 3A and Supplementary Dataset 5, 46 out of 58 LRP-DTGs show H3K9ac changes in either R1h, *pifq*, or in both). In addition, the correlation between H3K9ac and mRNA levels was slightly higher when only LRP-DTGs were considered (Pearson’s correlation, *r* = 0.68 for *pifq* vs. D, *r* = 0.81 for R1h vs. D and *r* = 0.86 for WLc vs. D, Supplementary Figure 5). In summary, these results show that H3K9ac changes occur in the majority of LRP-DTGs, in either R1h or *pifq*, suggesting that it could be a key regulatory factor in their transcriptional regulation.

### H3K9 Deacetylation Parallels Light-Triggered Transcriptional Repression and Apparently Precedes Transcriptional Induction

Given the close parallel between the transcriptional and chromatin responses, we approached the question of whether this represents a causal relationship by performing concurrent time-course analysis of the light-induced responses in these two parameters. For this purpose, we selected six PIF-induced/light-repressed LRP-DTGs (*PIL1*, *XTR7*, *HB2*, *CKX5*, *IAA19*, and *At5g02580*) and six PIF-repressed/light-induced LRP-DTGs (*RPT2*, *SIGE*, *LNK3*, *SPA1*, *BOH2*, and *At1g60590*), based on the extent of their changes in mRNA and H3K9ac levels in the genome-wide experiments, and on their potential key roles in red-light responsiveness based on their molecular function (Figures 3B,C, Supplementary Figure 6, and Supplementary Table 1).

**FIGURE 4:**
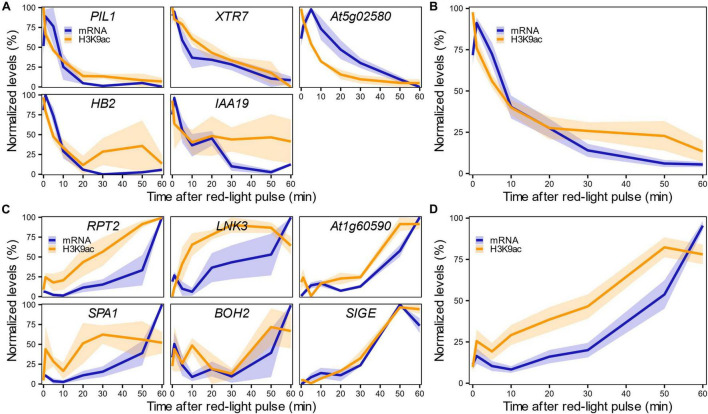
H3K9ac changes in LRP-DTGs parallel the rapid transcriptional response in light-repressed LRP-DTGs, and apparently precede transcription in light-induced LRP-DTGs. H3K9ac and mRNA changes measured by ChIP-qPCR and RT-qPCR in light-repressed **(A)** and light-induced **(C)** LRP-DTGs after a saturating red-light pulse. Averaged mRNA and H3K9ac levels for each group of genes are shown in **(B,D)**, respectively. Data were re-scaled to the minimum and maximum mRNA/H3K9ac values for each gene. Each colored line represents the averaged mRNA/H3K9ac levels at each time point and the shaded band represents the standard error of the mean (*n* = 3).

We first confirmed the results of the genome-wide analysis by measuring changes in H3K9ac levels in these 12 genes in response to R1h by ChIP-qPCR (Supplementary Figure 7). We then performed a detailed time-course analysis of the rapid changes in mRNA and H3K9ac levels over the 1-h period following initial light exposure. We measured both parameters by RT-qPCR and ChIP-qPCR in the 12 selected LRP-DTGs after a saturating red-light pulse. Highly variable mRNA and H3K9ac levels at the *CKX5* locus precluded any firm conclusions for this gene (Supplementary Figure 8). In broad terms, H3K9ac and mRNA levels were correlated (Supplementary Figure 9, Pearson’s correlation, *r* = 0.81). A more detailed analysis showed that most of the PIF-induced/light-repressed LRP-DTGs display rapid decreases in H3K9ac levels in response to red-light treatment, in parallel with the decrease in mRNA levels, declining sharply 5 min after the red-light pulse (Figures 4A,B). A converse pattern can be seen for the PIF-repressed/light-induced LRP-DTGs, where an increase in both mRNA and H3K9ac levels is detected shortly after red-light exposure (Figures 4C,D). However, in this case H3K9ac increase seems to occur earlier than mRNA induction, with detectable H3K9ac changes occurring around 10 min after the red-light pulse, while the mRNA increase happens 20 min after the red-light pulse (Figures 4B,D and Supplementary Figure 10).

These results suggest that H3K9ac deposition is necessary to initiate transcription, while its removal instantly triggers a reduction in steady state mRNA levels. It is also possible that this observed delay in transcription is caused by the fact that we are measuring steady-state levels of processed mRNA by RT-qPCR, and while this technique accurately measures mature mRNA levels of genes expressed in the dark (i.e., light-repressed LRP-DTGs), it might not capture the exact moment of light-induced LRP-DTGs transcriptional initiation.

### Real-Time Transcription Imaging Reveals an Earlier Timing of Light-Induced Transcription Initiation

In order to accurately measure a more continuous transcriptional readout and pinpoint the exact time of transcription initiation in response to light we generated several reporter lines. We first tested if we were able to recapitulate light-induced transcriptional initiation in transgenic lines expressing luciferase under the control of the *RPT2* promoter, a light-induced LRP-DTG (*pRPT2:LUC* lines). We chose *pRPT2* as it showed a strong and robust response to light in all our previous experiments. However, we were not able to detect any significant change in luminescence between *pRPT2:LUC* lines grown in the dark and after R1h treatment (Supplementary Figure 11A). This absence of response was not due to *pRPT2* lacking light/PIF responsive elements, as we could detect a large luminescence increase when we transformed this reporter into *pifq* background (Supplementary Figure 11B). It is likely that protein reporters that require transcription and translation in order to be measurable are not able to capture these short-term responses.

**FIGURE 5:**
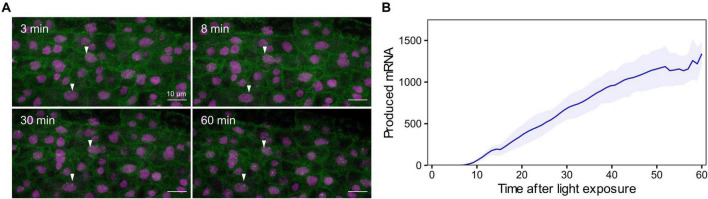
Single cell real-time transcription reveals an earlier start of light-induced *RPT2* transcription. **(A)** Maximum projection snapshots of *pRPT2:PP7* plants grown in true-dark conditions for 2 days. The time stamp indicates the time elapsed since the seedlings were exposed to light. Arrowheads point to the appearance of transcription spots. **(B)** Mean produced mRNA calculated as the integrated spot fluorescence over time. The colored line represents the average mRNA level and the shaded band represents the standard error of the mean. Data obtained from 2 to 5 technical replicates of four independent *pRPT2:PP7* transgenic lines are represented.

We then generated reporter lines in which we could measure mRNA transcription in real time (Supplementary Figure 12). The *RPT2* promoter was used to drive transcription of a mRNA tagged in its 5′ with a PP7 sequence recognized by a co-expressed GFP-tagged PP7 phage coat protein, enabling identification of nascent mRNA as fluorescent puncta (*pRPT2:PP7* – Supplementary Figure 12; Larson et al., 2011; Alamos et al., 2021). Imaging of dark-grown *pRPT2:PP7* lines exposed to light was able to recapitulate light-induced transcriptional initiation (Figure 5A). We measured real-time mRNA production in four independent *pRPT2:PP7* transgenic lines and we observed that transcription begins approximately 10 min after light exposure, slightly earlier than the increase in steady-state *RPT2* mRNA levels we detected by RT-qPCR (Figure 5B). The timing of transcription initiation coincides with the increase in H3K9ac levels observed by ChIP-qPCR (Figure 4C). Together, these results suggest that transcription initiation is accompanied by H3K9 acetylation, and within the time resolution limitations of our experiments, could indicate that they are intertwined processes. Development of more advanced techniques that measure chromatin changes with higher spatiotemporal resolution is required to better understand its relationship with transcriptional control.

### H3K9ac Writers and Erasers Involved in Long-Term Light Responses Do Not Participate in Global Fast Transcriptional Changes in Response to Light

**FIGURE 6:**
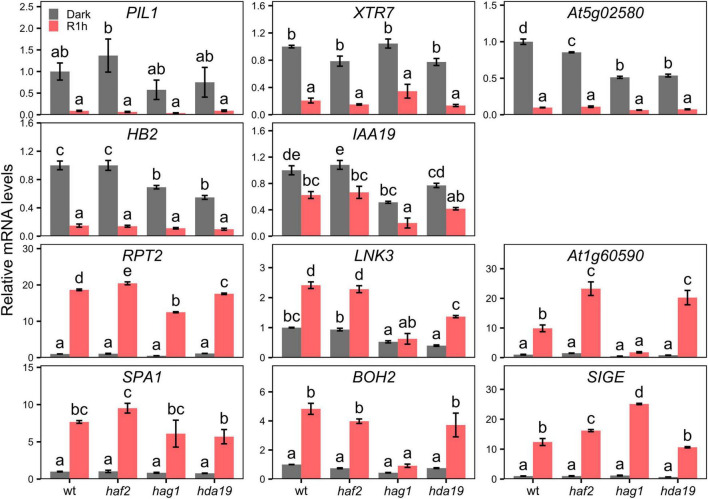
LRP-DTGs short-term transcriptional response to light is not generally affected in histone writer and eraser mutants. mRNA levels measured by RT-qPCR of LRP-DTGs in 3 days old seedlings grown in true dark or grown in the dark and then treated for 1 h with red light (R1h). mRNA levels are normalized to dark-grown wild-type. Error bars represent standard error of the mean. Statistical significance was determined by one-way ANOVA between each genotype and light treatment combination independently for each gene, with *post hoc* Tukey HSD test. Letters denote significant differences among means (*n* = 3).

It has been previously reported that the transcriptional program of light-grown plants is disrupted in mutants for H3K9ac deposition (HAG1/GCN5 and HAF2/TAF1) and removal (HDA19/HD1) (Bertrand et al., 2005; Benhamed et al., 2006; Guo et al., 2008). To test the possibility that these H3K9ac writer or eraser proteins could be involved in gene regulation shortly after initial light-exposure, we measured mRNA levels of the 12 selected LRP-DTGs in dark-grown and R1h-treated *hag1*, *haf2*, and *hda19* mutants. Contrary to what happens to other light-related genes in light-grown seedlings of these mutants, LRP-DTGs expression remained generally unaltered after a short red-light treatment. Although some mutants display a slight variation in R1h response when compared to the wild-type response, we only detected a completely R1h-insensitive responses on a few LRP-DTGs (*At1g60590*, *LNK3*, and *BOH2*), and only in the *hag1* mutants compared to wild type (Figure 6). These results indicate that HAF2 and HDA19 proteins, that are involved in H3K9ac regulation and previously identified to have a critical role in long-term light-induced chromatin states and transcription (i.e., plants grown under continuous white light), are not involved in general short-term transcriptional regulation in response to light. HAG1 seems to control expression of only a small subset of LRP-DTGs. Alternatively, another possible explanation is that because of functional redundancy of histone modifiers (Pandey et al., 2002), mutation in a single family member is not sufficient to affect the LRP-DTGs rapid transcriptional response to light. It remains to be explored which other possible factors might be responsible for the quick changes in H3K9ac in response to light exposure.

## Conclusion

The aim of the present study was to identify key genes that respond in the first instance to the first light exposure in plants, and understand the factors involved in their regulation. To do this, we focused on the red-light phytochrome signaling pathway. For this purpose, we focused on known PIF direct-target genes. We found that only a subset of the previously described PIF-DTGs respond most rapidly to light exposure. We redefined them as LRP-DTGs. These genes probably are the most direct targets of PIF regulation and play a key role in photomorphogenesis initiation. We found that the majority of these genes also show very rapid changes in H3K9ac levels, that accompany their transcriptional response, in the very initial minutes after light exposure. These findings suggest that chromatin remodeling is mechanistically linked to the very initial light response of these genes. The fact that H3K9ac is present in repressed genes, albeit at low levels, raises an interesting hypothesis: do basal H3K9ac levels prime specific genes so they can rapidly respond to changing light conditions? More precise chromatin state measuring techniques coupled with chromatin state-altering treatments are required to characterize these changes. This study contributes to our understanding of transcriptional regulation in response to environmental changes, and describes a system in which chromatin dynamics in response to environmental cues can be analyzed. The precise mechanism of how these histone modifications are performed and how they interact with other factors to orchestrate this response remain to be elucidated.

## Materials and Methods

### Plant Materials and Growth Conditions

The Columbia-0 ecotype of *Arabidopsis thaliana* was used for all experiments. *pif1pif3pif4pif5* (*pifq*) line is described in Leivar et al. (2008), *hag1-5* (SALK_048427) in Kornet and Scheres (2009), *haf2-5* (SAIL_548_G03) in Lee and Seo (2018), and *hda19-4* (SALK_139443) in Kim et al. (2008). To generate *pRPT2:LUC* lines, 3342 bp upstream of the RPT2 start codon were amplified by polymerase chain reaction (PCR) with primers *Bam*HI-pRPT2-5/pRPT2-3-*Pst*I and cloned into the pC1302-35S:RLUC vector (Zhang et al., 2013). To generate *pRPT2:PP7* lines, 3356 bp upstream of the RPT2 start codon were amplified by PCR with primers 13Rb-RPT2F and 13Rb-RPT2R and cloned into the AL13Rb plasmid (Addgene # 161006) (Alamos et al., 2021). Primers are described in Supplementary Dataset 9. Constructs were transformed into Arabidopsis by the floral dip method (Clough and Bent, 1998).

Plants were germinated in true-dark conditions as described in Leivar et al. (2008). Briefly, seeds were sterilized, plated in MS medium without sucrose under white light and stratified for 4 days at 4°C in darkness. Afterward, they were irradiated for 3 h with white light to induce germination, followed by a saturating 5 min far-red light pulse. They were grown in the dark at 21°C for 2 days before being collected (D samples) or grown in the dark for 47 h and treated with 10 μE⋅m^–2^⋅s^–1^ red light for 1 h (R1h samples). White-light grown samples (WLc) were grown under continuous white-light (100 μE⋅m^–2^⋅s^–1^ PAR) after stratification for 2 days. For the time-course experiments, a saturating red-light pulse (5000 μE in 1 min) was given after 2 days in the dark and samples were collected at the indicated time-points.

### RNA Sequencing

Total RNA from 2-day old “true dark”-grown (D), R1h and WLc treated seedlings was extracted and processed as described in Zhang et al. (2013). RNA was extracted from 100 mg of ground tissue using RNeasy Plant Mini Kit (Qiagen cat. 74904) using RNase-Free DNase (Qiagen cat. 79254) following manufacturer instructions. The sequencing library construction was adapted from 3′-end RNA-seq protocol (Yoon and Brem, 2010) and performed as described in Zhang et al. (2013). The size of purified library DNA was validated by Bioanalyzer 2000. Libraries were assayed by 50-cycle single-end sequencing on the HiSeq 2000 platform. Sequencing reads were aligned to the TAIR9 representative transcriptome using Bowtie (Langmead et al., 2009) with zero mismatches allowed. Only reads mapping uniquely to the 3′-end 500-bp region of the coding strand were counted for gene expression. Differentially expressed genes were identified using the edgeR package (Robinson et al., 2010), and genes with statistically significant two-fold (or greater) mRNA changes were defined as those that differ by ≥2-fold with an adjusted *P* value ≤ 0.05. Sequencing data have been deposited in NCBI’s Gene Expression Omnibus (Edgar et al., 2002) and are accessible through GEO Series accession number GSE181167. Previously published sequencing data can be accessed through GEO Series accession number GSE39217.

### Chromatin Immunoprecipitation and Sequencing

For each replicate, 2.5 gr of 2-day old wild-type and *pifq* D, R1h and WLc treated seedlings were processed as described in Gendrel et al. (2005) using 5 μgr Diagenode Ab C15410004 H3K9ac antibody and a mix of 25/25 μl of protein-A/G Dynabeads (Invitrogen). Samples were collected and processed under a green safelight. 5–10 ng of DNA per sample were used for library preparation with Accel-NGS 2S Plus DNA Library Kit (Swift Biosciences) and 9 PCR cycles. 300–700 bp fragments were purified and sequenced in an Illumina HiSeq 4000 by SR50 Single Read Sequencing. Reads were aligned to Arabidopsis thaliana TAIR9 reference genome using Bowtie2 (Langmead and Salzberg, 2012). H3K9ac enriched regions were identified using the BayesPeak algorithm with lower-bound summarization method (Spyrou et al., 2009). Differential enrichment analysis was performed with DiffBind (Stark and Brown, 2011), comparing H3K9ac enrichment in these regions among different samples. Only peaks with statistically significant different levels with an FDR ≤ 0.05 were used in the analysis. Each peak was assigned to the gene with the nearest transcriptional start site using the TAIR9 gene annotation. Sequencing data have been deposited in NCBI’s Gene Expression Omnibus (Edgar et al., 2002) and are accessible through GEO Series accession number GSE181432.

### Chromatin Immunoprecipitation Time-Course

Due to the time-sensitive nature of the time course experiment, crosslinking time was reduced to 5 min. Additionally, due to the impossibility to process 27 samples simultaneously, to reduce sample-batch variability as much as possible, we collected samples in batches of three always collecting three different randomized time points each day. We prepared the samples (sowing the seeds and true-dark treatment) and collected them at the same time of the day to avoid any circadian clock effect.

### Reverse Transcription Quantitative Polymerase Chain Reaction

Total RNA was extracted from 100 mg of dark grown or R1h treated seedlings per biological replicate, and three biological replicates were used per time point using RNeasy Plant Mini Kit (Qiagen cat. 74904) with RNase-Free DNase (Qiagen cat. 79254) following manufacturer instructions. One μgr of RNA was used to make cDNA with the High-Capacity cDNA archive kit (Thermo Fisher Scientific cat. 4368814). qPCR reactions were performed with SYBR Green Master Mix (Thermo Fisher Scientific cat. 4309155) using three technical replicates per reaction in a CFX96 Touch Real-Time PCR Detection System (Bio-Rad). *TUBULIN1* was used as a reference for ChIP-qPCR experiments and *PP2AA3* for RT-qPCR (Czechowski et al., 2005). Primers are described in Supplementary Dataset 9.

### Luciferase Assays

Approximately 100 mg of 2-day old seedlings were collected and ground in liquid nitrogen. Total protein was extracted using 100 μl of Passive Lysis Buffer (Promega). Twenty microliters of the supernatant were used to measure the LUC and RLUC activity using a Dual-Luciferase Reporter Assay System (Promega) according to the manufacturer’s instructions in a Tecan M-100PRO plate reader. Firefly luminescence was normalized by the constitutively expressed Renilla luciferase internal control.

### Real-Time Transcription Imaging

Image acquisition and analysis was performed as described in Alamos et al. (2021). Estimation of mRNA production was calculated as described in Garcia et al. (2013) and Alamos et al. (2021). The Leica SP8 microscope was operated using the LASX software in manual mode. Data was taken at a constant laser power for the 488 nm laser and the 569 nm laser throughout the duration of a single time course experiment and across experiments. No auto correction or manipulation was done during acquisition, except for adjustment of the z position due to sample drift. Each channel -GFP and mScarlet- was acquired using a different HyD detector and treated independently throughout the downstream analysis. The imaging setup was as follows: 63× magnification; zoom 2; image size 1024 × 512 × 25 z slices; pixel xy size 90 nm; z slice depth 500 nm; bidirectional scanning at 300 Hz; pinhole 1 airy unit at 500 nm; no frame or line average; no frame accumulation, line accumulation 3; 488 nm at ∼5% laser power (calibrated to μW) and 569 nm at 1.5% laser power, frame rate 1 min/stack.

## Data Availability Statement

The datasets presented in this study can be found in online repositories. The names of the repository/repositories and accession number(s) can be found in the article/Supplementary Material.

## Author Contributions

EG-G and PQ conceived the project and wrote the manuscript. EG-G, SÁ, YZ, KN, HG, and PQ designed the experiments. EG-G, YZ, SÁ, and JD-R performed the experiments. EG-G and SÁ performed the data analysis. All authors have edited and commented on the manuscript and have given their approval of the final version.

## Conflict of Interest

The authors declare that the research was conducted in the absence of any commercial or financial relationships that could be construed as a potential conflict of interest.

## Publisher’s Note

All claims expressed in this article are solely those of the authors and do not necessarily represent those of their affiliated organizations, or those of the publisher, the editors and the reviewers. Any product that may be evaluated in this article, or claim that may be made by its manufacturer, is not guaranteed or endorsed by the publisher.
