# Supplementary material for: Chromatin Changes in Phytochrome Interacting Factor-Regulated Genes Parallel Their Rapid Transcriptional Response to Light
Source: Front Plant Sci. 2022 Feb 17;13:803441. doi: 10.3389/fpls.2022.803441 (PMC8891703; doi:10.3389/fpls.2022.803441)
Supplement: Supplementary file 2 [file Presentation_1.pdf]

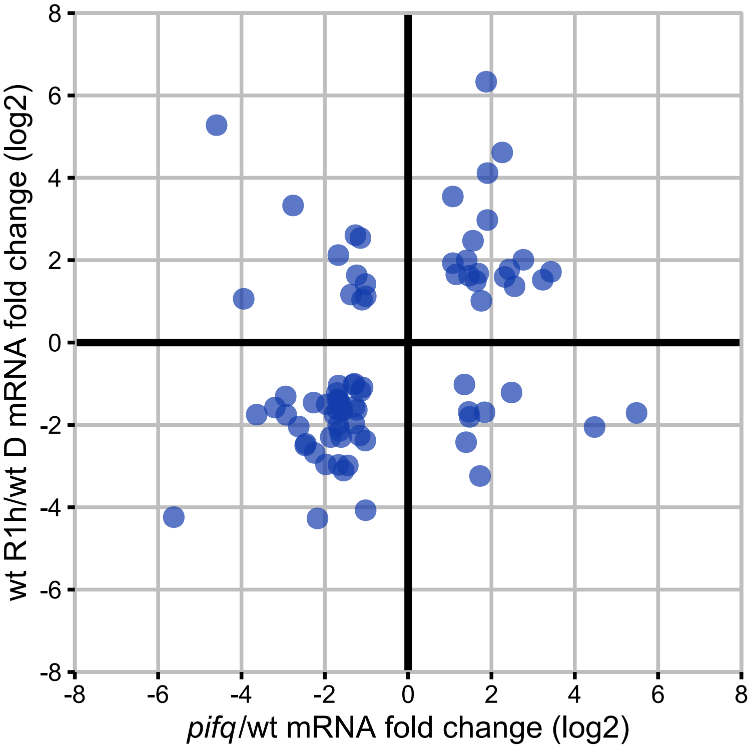

Figure S1. Only a subset of genes previously defined as PIF-DTGs in dark-grown wild-type seedlings respond after exposure to red light for one hour. Scatter plot showing the fold change in mRNA levels of previously defined PIF-DTGs between dark-grown *pifq* and wild type plants compared to wild type plants treated with 1 hour of red light versus dark-grown wild type plants. Only 78 of the 338 previously defined PIF-DTGs show a statistically significant two-fold change after 1h of red light treatment (Supplemental Dataset S5). Statistically significant (FDR < 0.05) two-fold change values are plotted.

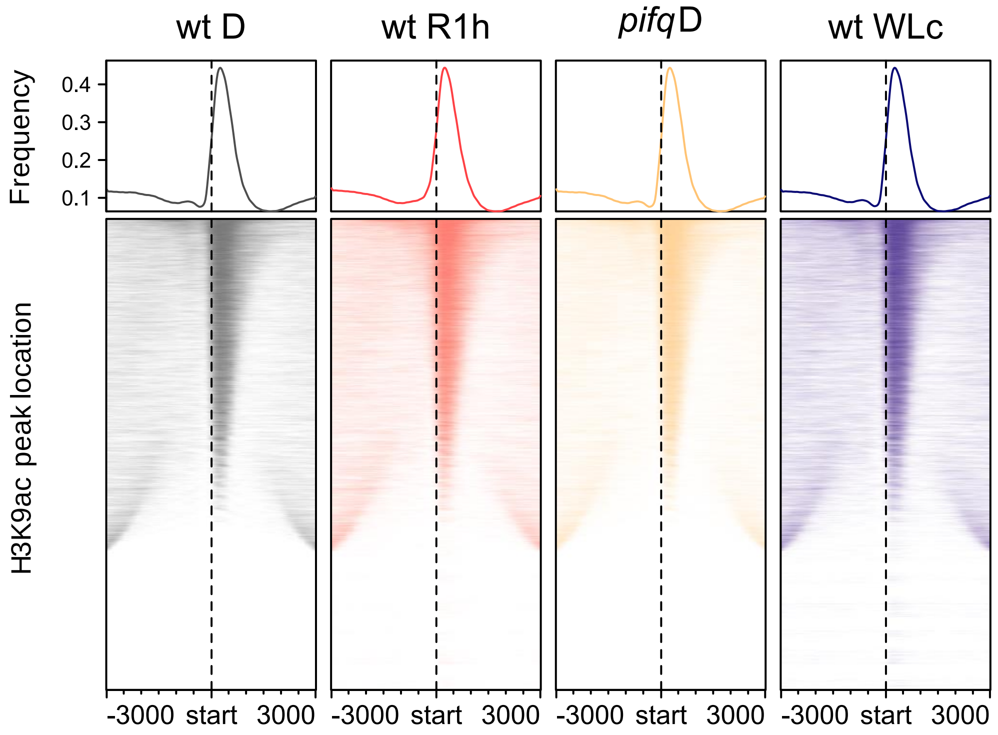

Figure S2. H3K9ac peaks localize downstream of the transcriptional start site. Profile of H3K9ac peak location identified by Bayes Peak in wt D, wt R1h and *pifq* D, and wt WLc. Top panel represents the frequency of the peak location while the bottom panel represents the location of H3K9ac peaks respective of the transcriptional start site (3000 base pairs upstream and downstream).

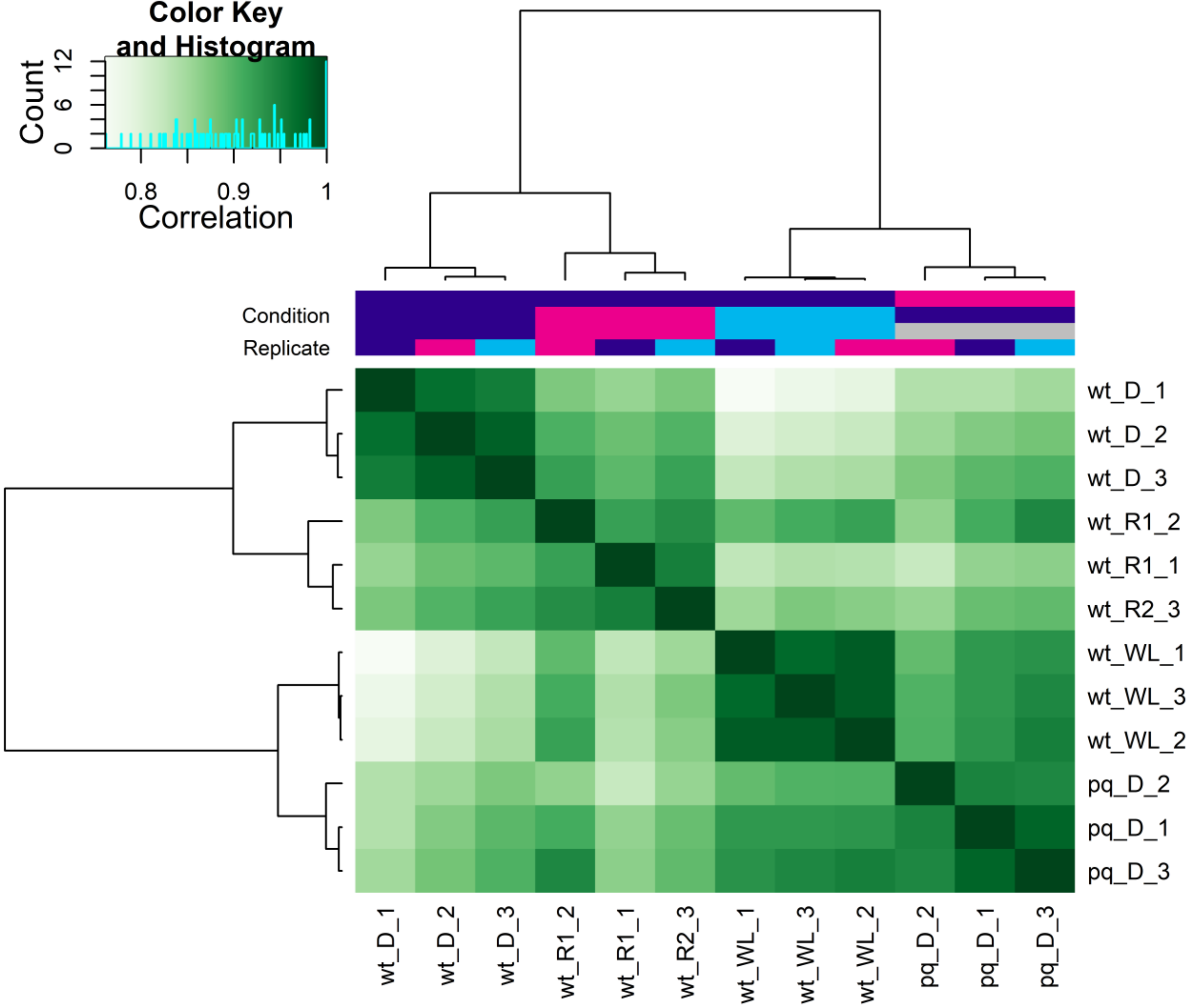

Figure S3. Genome-wide H3K9ac profile of *pifq* plants grown in the dark is similar to that of white-light grown plants. Correlation heatmap, using genome-wide H3K9ac occupancy (peak caller score) of significant binding peaks called by BayesPeak. wt, wildtype; pq, *pifq*; D, dark-grown; R1, dark-grown and treated with red-light for one hour; WL, grown in continuous white light; numbers 1 to 3 represent biological replicates.

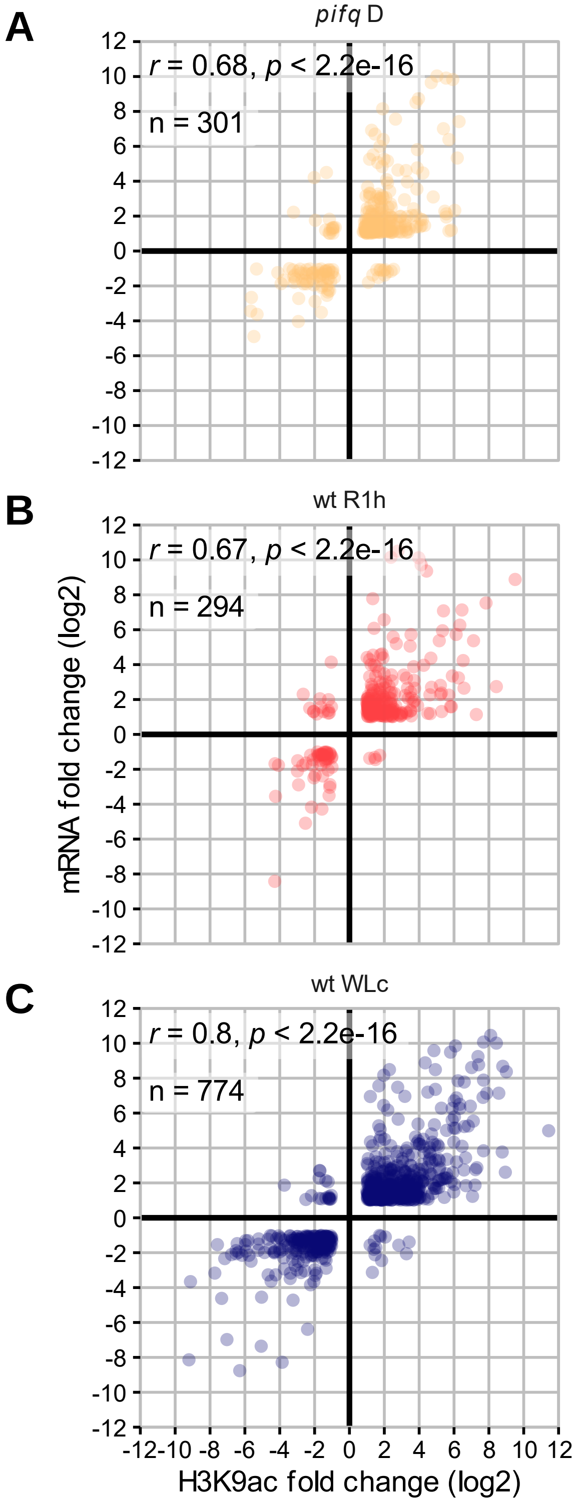

Figure S4. H3K9ac changes correlate with steady state mRNA levels. Scatter plots showing the correlation between H3K9ac and mRNA changes in the ChIP-seq experiments comparing dark-grown *pifq* vs wild type (A), 1h red light treatment vs dark-grown wild type (B) and white light grown vs dark-grown wild-type seedlings. Only statistically significant (FDR < 0.05) two-fold change values were selected. r, Pearson's correlation coefficient; p, p-value.

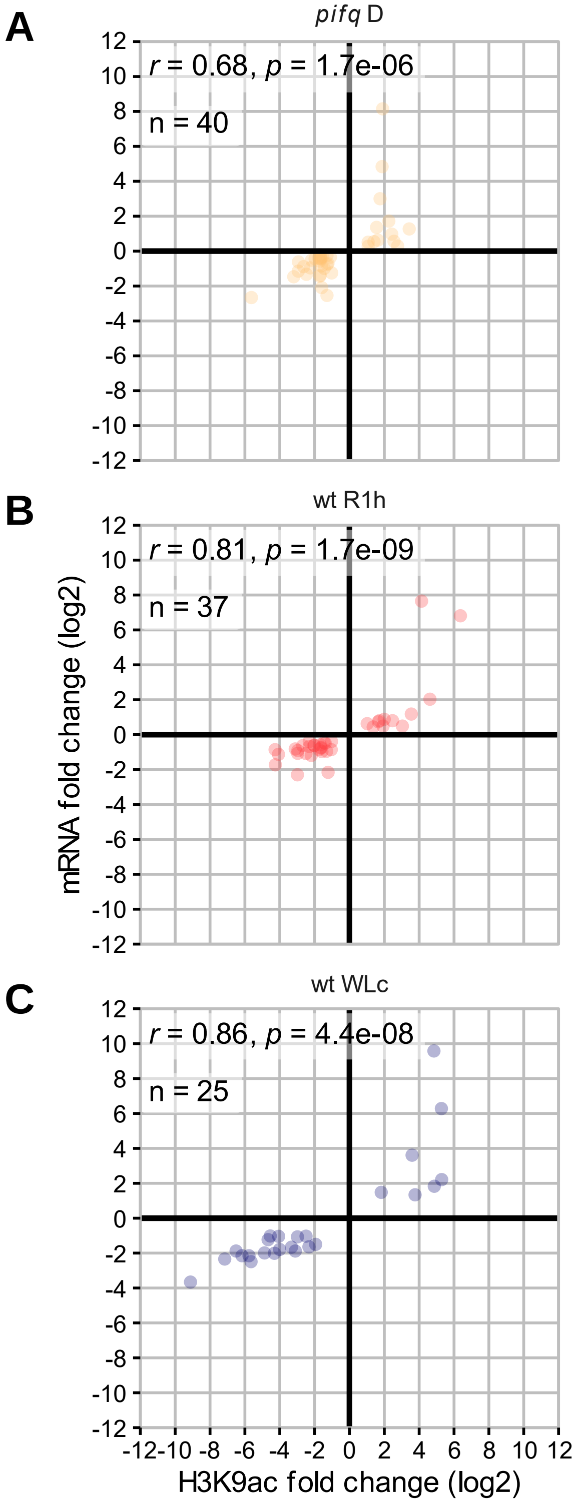

Figure S5. H3K9ac changes correlate with steady state mRNA levels in LR-PIF-DTGs. Scatter plots showing the correlation between H3K9ac changes and mRNA changes in LR-PIF-DTGs in the ChIP-seq experiments comparing dark-grown *pifq* vs wild type (A), 1h red light treatment vs dark-grown wild type (B) and white light grown vs dark-grown wild-type seedlings. Only statistically significant (FDR < 0.05) two-fold change values were selected. r, Pearson's correlation coefficient; p, p-value.

Locus	Gene	Regulated by PIF	logFC *pifq*/wt	logFC wtR1/wtD
AT2G46970	phytochrome interacting factor 3-like 1 (PIL1)	1345	-5.58	-4.24
AT5G02580	Plant protein 1589 of unknown function	15	-1.41	-2.98
AT4G16780	ARABIDOPSIS THALIANA HOMEOBOX PROTEIN 2 (HB2)	1345	-1.97	-2.96
AT4G14130	xyloglucan endotransglucosylase/hydrolase 15 (XTR7/XTH15)	135	-2.43	-2.50
AT3G15540	indole-3-acetic acid inducible 19 (IAA19)	14	-1.64	-1.46
AT1G75450	cytokinin oxidase 5 (CKX5)	1	-1.68	-1.38
AT5G52570	beta-carotene hydroxylase 2 (BETA-OHASE 2)	15	2.48	1.36
AT3G12320	NIGHT LIGHT-INDUCIBLE AND CLOCK-REGULATED 3 (LNK3)	15	1.44	2.00
AT2G46340	SUPPRESSOR OF PHYA-105 1 (SPA1)	1	1.06	3.55
AT1G60590	Pectin lyase-like superfamily protein	5	1.89	4.11
AT2G30520	ROOT PHOTOTROPISM 2 (RPT2)	5	2.20	4.62
AT5G24120	sigma factor E (SIGE)	1	1.80	6.33

Table S1. LRP-DTGs selected for further analysis. Six light-repressed/PIF-induced LRP-DTGs and six light-repressed/PIF-induced LRP-DTGs were selected based on their H3K9ac fold change and functional relevance for a more detailed analysis. The “Regulated by PIF” column specifies which PIF(s) regulate expression of the gene based on the criteria defined in Pfeiffer et al., 2014. logFC refers to log2 fold-change between the two conditions.

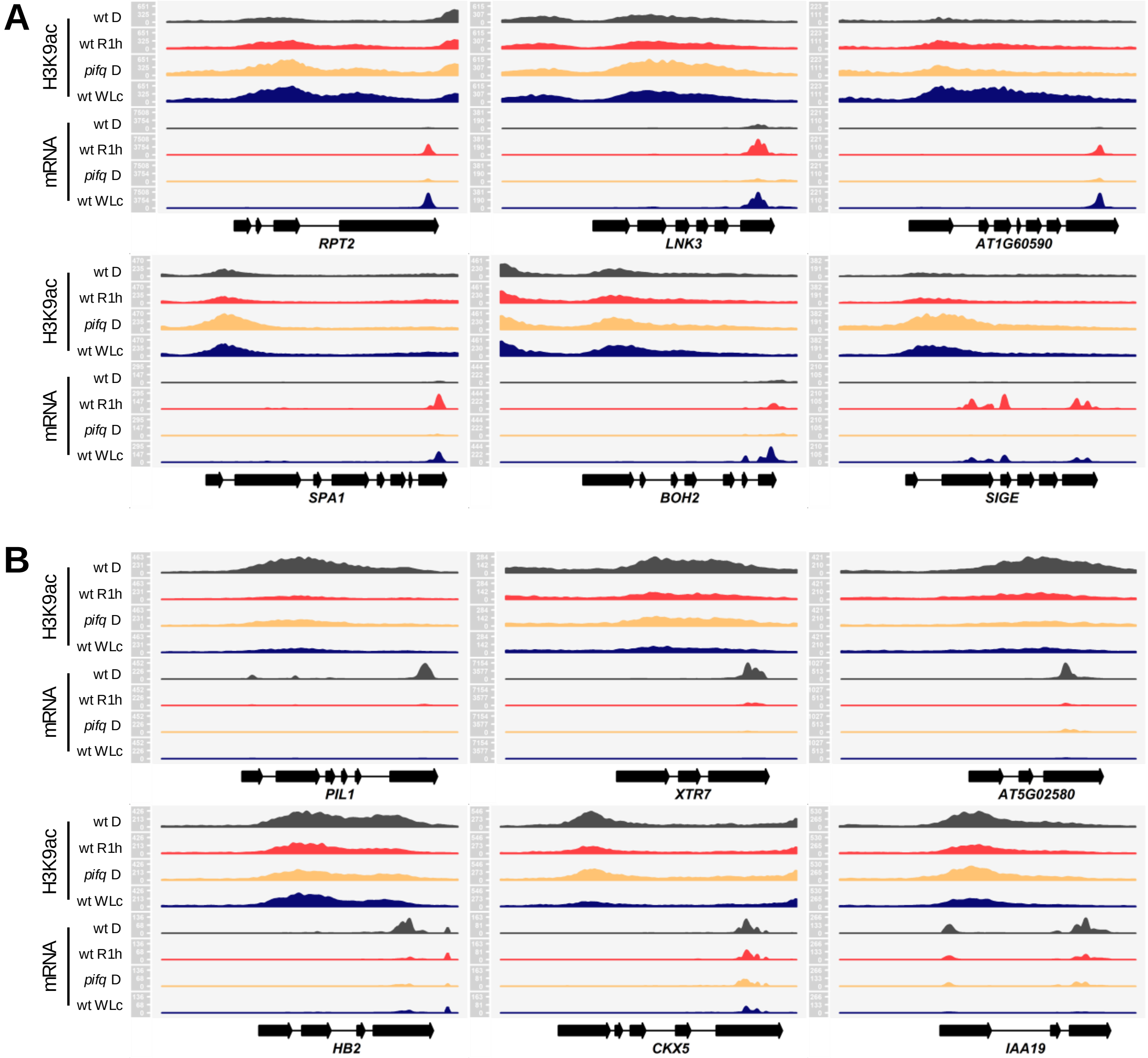

Figure S6. H3K9ac levels change in light-responsive PIF-DTGs (LRP-DTGs) both in *pifq* and in response to red light. A, Read mapping profile of H3K9ac ChIP-seq and RNA-seq in light-induced/PIF-repressed LRP-DTGs. B, Read mapping profile of H3K9ac ChIP-seq and RNA-seq in light-repressed/PIF-induced LRP-DTGs. For each gene, 1000 bp upstream and 250 bp downstream of their representative transcript are shown. Read count is scaled independently for each gene, and for mRNA and H3K9ac levels. RNA-seq data from *pifq* seedlings and their corresponding wild-type control were obtained from Zhang et al., 2013. Note that RNA-seq was performed on 3'-end purified mRNA.

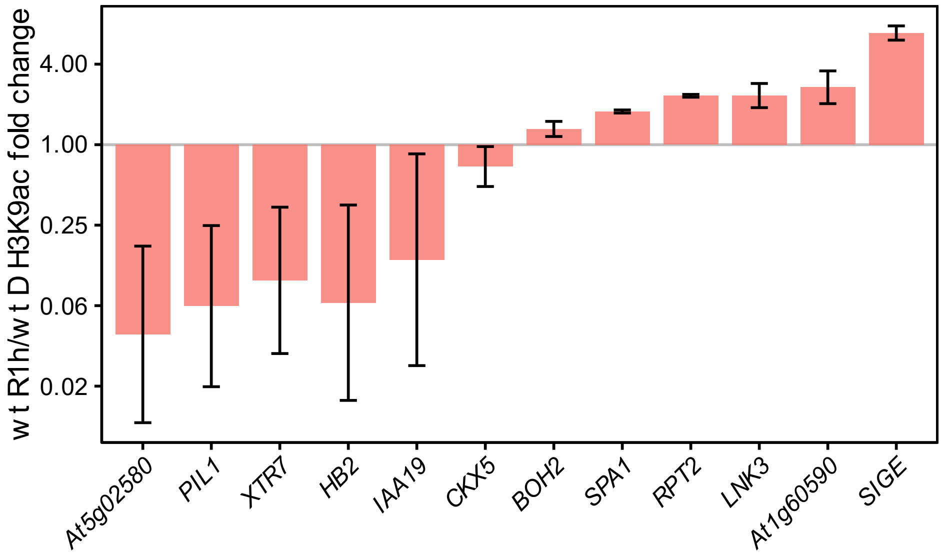

Figure S7. H3K9ac levels change in light-responsive PIF-DTGs (LRP-DTGs) in response to red light. H3K9ac levels measured by ChIP-qPCR in six induced and six repressed PIF-DTGs comparing dark-grown vs R1h wildtype samples. Error bars represent standard error of the mean.

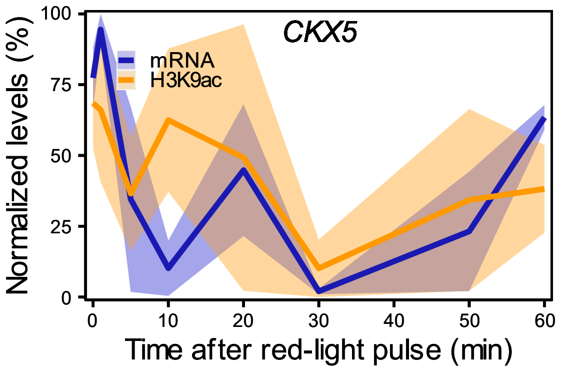

Figure S8. Highly variable mRNA and H3K9ac levels in the *CKX5* locus. H3K9ac and mRNA changes measured by ChIP-qPCR and RT-qPCR in *CKX5*, a light-repressed LRP-DTG after a saturating red light pulse. Data were re-scaled to the minimum and maximum mRNA/H3K9ac values. Each colored line represents the averaged mRNA/H3K9ac levels at each time point and the shaded band represents the standard error of the mean (n = 3).

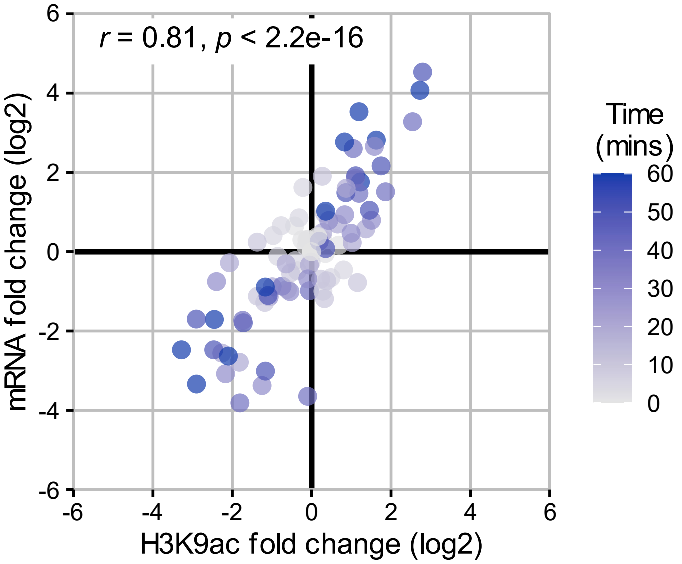

Figure S9. H3K9ac correlation with mRNA levels. Scatter plot showing the correlation between H3K9ac and mRNA levels in the samples used for the time-course experiment (Figure 4). Fold change values are normalized to the 0 minutes time point for each gene. r, Pearson's correlation coefficient; p, p-value.

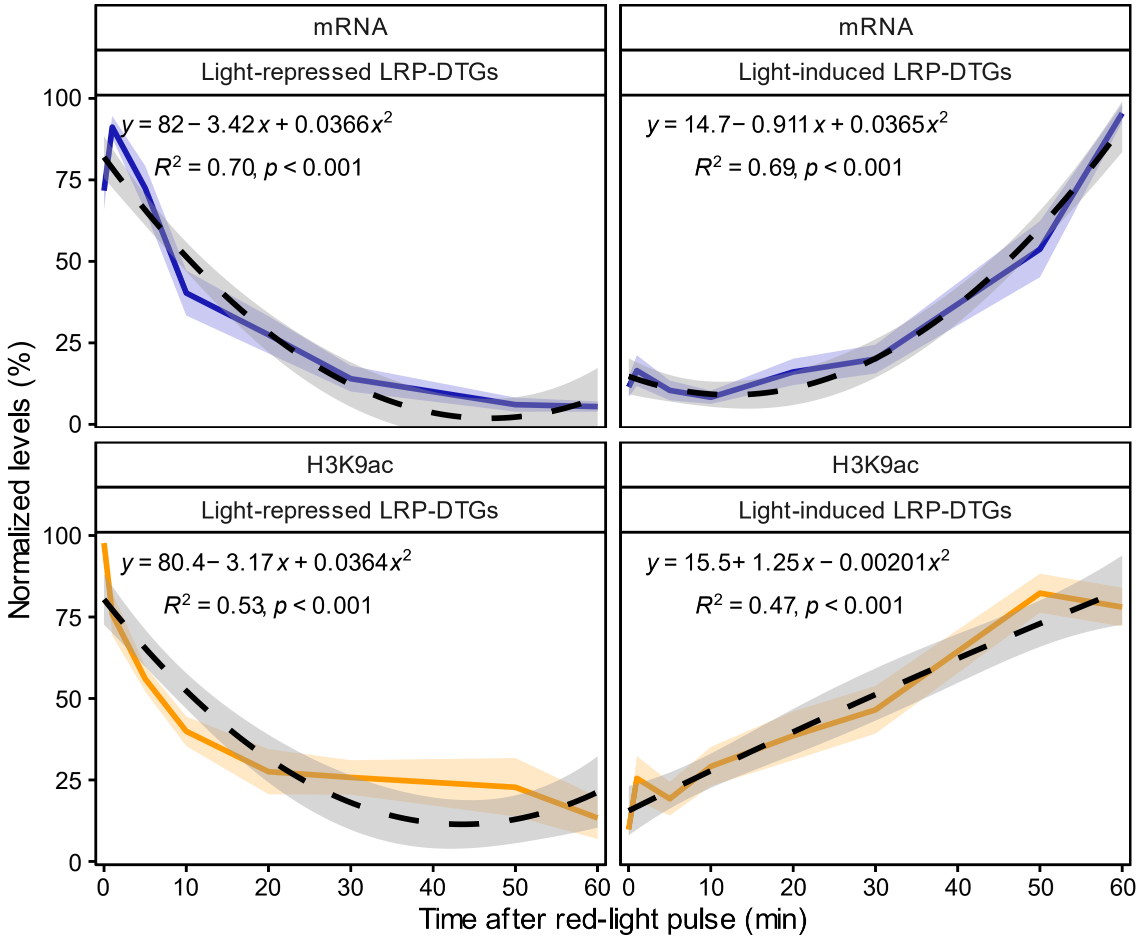

Figure S10. mRNA and H3K9ac trajectories are very similar in light-repressed LRP-DTGs while they differ in light-induced LRP-DTGs. mRNA and H3K9ac data were fitted by linear regression to a quadratic function separately for light-repressed and light-induced LRP-DTGs. In light-repressed LRP-DTGs, H3K9ac and mRNA rate of change is quadratic. In light-induced LRP-DTGs the rate of change for H3K9ac is almost linear, while mRNA change is still quadratic. These results corroborate that H3K9ac changes earlier than mRNA in light-induced LRP-DTGs. For light-repressed LRP-DTGs mRNA and H3K9ac linear fits, R^2=0.99 (p-value = 7.59e-08), for light-induced LRP-DTGs mRNA and H3K9ac linear fits, R^2=0.8 (p-value = 0.002727). R^2, coefficient of determination; p, p-value.

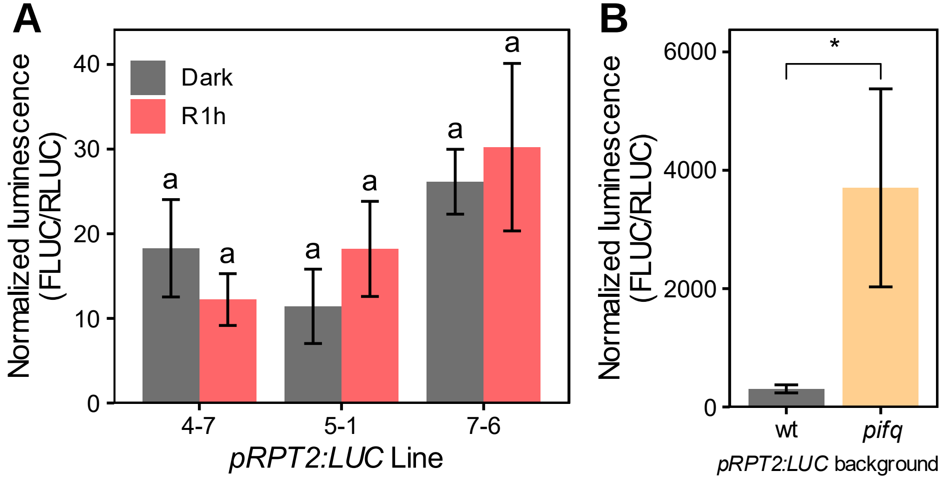

Figure S11. Luciferase reporter lines only recapture *RPT2* regulation by PIFs, not its rapid transcriptional response to light. A, Luminescence measured in three independent homozygous *pRPT2:LUCIFERASE* lines grown in true dark or true dark plus 1 h red light (n = 3). B, Luminescence measured in *pRPT2:LUCIFERASE* lines in wild-type or *pifq* genetic background grown in true dark (n = 5 independent lines for each genotype). Luminescence from Firefly luciferase (FLUC) under transcriptional control of *RPT2* promoter is normalized by constitutively expressed Renilla luciferase activity (RLUC). Error bars represent standard error of the mean. Statistical significance in A was determined by one-way ANOVA with post-hoc Tukey HSD test. Letters denote significant differences among means. Statistical significance in B was determined by T-test, *, p-val < 0.05.

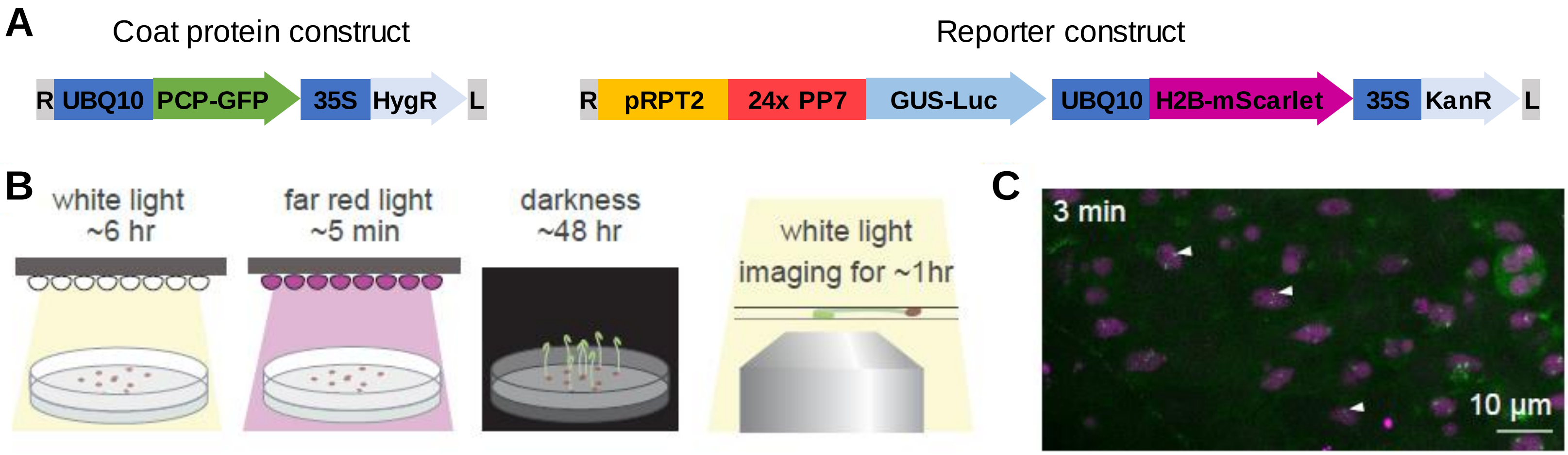

Figure S12. Experimental setup for pRPT2:24xPP7 lines. A, Diagram of the T-DNA constructs used to obtain *pRPT2:PP7* experimental lines (adapted from Álamos et al., 2021). R and L, T-DNA border sequences; UBQ10, *UBIQUITIN10* promoter; PCP-GFP, GFP-tagged phage PP7 coat protein; 35S, 35S cauliflower mosaic virus promoter; HygR, Hygromycin resistance gene; pRPT2, 3329 bp upstream of *RPT2* gene; 24x PP7, 24 repeats of the PP7 phage RNA sequence recognized by PCP; GUS-Luc, β-glucuronidase-Luciferase fusion; H2B-mScarlet, Histone 2B fused to mScarlet as nuclear marker; KanR, Kanamycin resistance gene. B, Experimental setup used for imaging. True dark light regime used. C, Maximum projection snapshot of a cotyledon from a *pRPT2:PP7* plant grown in white light.
